# Dr. Ira F. Hart

**Published:** 1915-01

**Authors:** 


**Dr. Ira F. Hart,** Jefferson 1852, died at his home in Elmira October 30, 1914, aged 84. He was a Surgeon of Volunteers during the entire Civil War, and was subsequently editor of the Saturday Evening Review and of the Elmira Advertiser.

